# Association between gender-based violence and hypertension among women in the Kyrgyzstan Republic

**DOI:** 10.1186/s12872-022-02970-6

**Published:** 2022-12-15

**Authors:** Mustapha S. Abba, Chidozie U. Nduka, Seun Anjorin, Olalekan A. Uthman

**Affiliations:** 1 Division of Health Sciences, Warwick Medical School, The University of Warwick, Coventry, CV4 7AL UK; 2 Warwick Centre for Global Health, Division of Health Sciences, Warwick Medical School, The University of Warwick, Coventry, CV4 7AL UK; 3 Division of Epidemiology and Biostatistics, Department of Global Health, Stellenbosch University, Stellenbosch, South Africa; 4 Department of Public Health (IHCAR), Karolinska Institutet, Stockholm, Sweden

**Keywords:** Hypertension, Gender-based violence, Domestic violence, Intimate partner violence, Kyrgyz Republic

## Abstract

**Background:**

Gender-based violence (GBV) is a significant global public health problem and the most prominent human rights violation severely impacting women’s health and wellbeing. Therefore, the aim of this study is to evaluate the association between gender-based violence and hypertension in Kyrgyzstan Republic.

**Methods:**

This study was conducted using population-based data of women from the 2018 Kyrgyzstan Demographic and Health Survey. The odds ratio was calculated to measure the association between GBV and hypertension, and p-values < 0.005 was considered statistically significant.

**Result:**

We included data of 4793 participants, and 621 (13%) of them had hypertension. Participants exposed to GBV were 24% more likely to have hypertension than unexposed participants (OR = 1.24, 95% CI: 1.03–1.48). Of all women with hypertension, 206 (33.0%) were exposed to GBV. Participants with secondary education or higher exposed to GBV were 24% more likely to be hypertensive than GBV unexposed women with the same education levels OR = 1.24, 95% CI: 1.04–1.49). Unemployed participants exposed to GBV were 45% more likely to develop hypertension than their unexposed counterparts (OR = 1.45, 95%CI: 1.15–1.81). Rural residents exposed to GBV were also 29% more likely to have hypertension than those unexposed to GBV (OR = 1.29, 95% CI: 1.04–1.59). The odds of hypertension among those exposed to GBV increase with age.

**Conclusion:**

The study revealed that GBV is a significant factor of having hypertension among Kyrgyz women.

## Background

Gender-based violence (GBV) against women is a significant public health problem affecting their quality of life [1], and the most prevalent human rights violation globally [2]. GBV is a dehumanising, pervasive and oppressive form of violence and intimate partner violence (IPV) is the most significant component of GBV [3]. The United Nations Universal Declaration of Human rights stipulates that “*All human beings are born free and equal in dignity and rights*” *and* “*everyone has the right to life, liberty and security of person*” [4]*.* Despite the international commitment to achieve gender equality, one in three women experience physical or sexual violence in their lifetime, mainly by an intimate partner, especially in low- and middle-income countries (LMICs) [5] and the prevalence varies between and within regions and countries and even between neighbourhoods [1, 6]. Southern Asia and Sub-Saharan Africa have the highest prevalence rates of IPV among women aged 15–49, ranging from 33 to 51% [7], including the Kyrgyz Republic and other South Asian countries. In Kyrgyzstan, the high IPV prevalence may be attributed to the traditional practice called “ala kachu”, which is bride kidnapping [8, 9]. Women who experience violence are more likely to face physical, sexual, reproductive, and mental health consequences [7]. GBV victims are more likely to suffer severely from depression and post-traumatic stress disorder, which are known risk factors for hypertension [10]. Gilbert et al. demonstrated the impact of GBV on drug abuse among Kyrgyz women, where most of them reported experiencing physical abuse or sexual IPV (73%), and physical abuse (60%) [11]. Illicit drugs are associated with elevated blood pressure, making GBV victims who also used illicit drugs more at risk for hypertension [12].

A study conducted on young South African women aged 15–34 years found that 68% of the women with hypertension had experienced IPV [13]. The odds of developing hypertension increase if women experience physical or sexual violence. GBV has several adverse health consequences, but little is known about its association with hypertension in Kyrgyzstan, where the burden of hypertension is equally high and underreported [14]. Therefore, this study aimed to provide more evidence by examining the association between the experience of GBV and the risk of having hypertension among women in Kyrgyzstan.

## Methods

### Study design

This study was based on secondary analyses of population-based data from the 2018 Kyrgyzstan Demographic and Health Survey (DHS). The DHS collected data on demographic, environmental, socio-economic, and health issues, including GBV and hypertension. The sample for the 2018 Kyrgyzstan DHS was nationally representative covering the entire population in the country on individual and household levels. The detailed survey design and procedures are found in the 2018 Kyrgyzstan DHS report.

During the DHS survey 6,021 women aged 15–49 years were interviewed in the GBV module of the DHS and 4793 women were screened for hypertension.

### Eligibility criteria and sampling

We included all women aged 15–54 years who responded to the GBV DHS questions and whose blood pressure levels were sampled.

### Outcome variable

The study’s primary outcome was hypertension. Respondents were deemed to have hypertension if their systolic or diastolic blood pressure was greater than 140 mm Hg or 90 mm Hg, respectively, or if they were currently using an anti-hypertensive medication. According to the respondent’s cuff size, the blood pressure was measured three times at intervals of ten minutes using small, medium, and large cuff sizes. To classify hypertension, the average of the second and third measures was employed [15].

### Exposure variable

Using a modified and previously validated version of the Conflict Tactic Scale [16], exposure to GBV/IPV against women (spousal physical, sexual, and emotional abuse) was evaluated. GBV is defined as exposure to one or more of the following situations perpetrated by a husband or partner ever. To determine physical abuse, six factors were considered: whether the abuser ever shoved, shook, or threw something; whether they ever slapped; whether they ever used harmful punches; whether they ever kicked or dragged; whether they ever attempted to strangle or burn; and whether they ever used a knife, gun, or other weapon to threaten the other. Forced sexual contact and other unwanted sexual activities were the two characteristics utilised to quantify sexual abuse. The following three factors were used to gauge emotional abuse: whether or not her partner had ever made her feel bad or embarrassed in front of others, threatened to hurt her, or insulted her [17].

### Control variables

The following individual-level factors were included in the study: occupation (working or not working); residential area (urban or rural); Respondents’ age in years, educational attainment (no education, primary, secondary or higher); weight was measured to the nearest 0.5 kg with subjects in light clothing, and height was measured to the nearest 0.1 cm. Body mass index (BMI) was calculated and categorized as underweight (< 18.5 kg/m^2^), overweight (25–29.9 kg/m^2^), and obese (≥ 30 kg/m^2^). DHS wealth index was used as a proxy indicator for the socio-economic position. Thus, wealth status was categorised into poorest, poorer, middle, richer and richest categories [18].

### Statistical analysis

In the descriptive statistics, the distribution of respondents by the key variables was expressed as frequencies and percentages. We used Pearson’s chi-squared test to compare participants’ characteristics and hypertension. Multivariate logistic regression analysis was also used to identify the association between GBV and hypertension. Odds ratios (OR) and 95% confidence interval (CI) were calculated to measure the strengths of associations, and a statistical significance was set at the p-value < 0.05. Data analysis was performed using Stata software (Version 17, StataCorp, Texas, USA).

## Results

Our study included 4793 women, mostly aged 25–34 years (39.1%), followed by those aged 35–44 years. Most of the respondents had secondary or higher education (99.7%), and about 18.1% and 19.5% of participants belonged to the richest and richer wealth quintile, respectively. The majority (62.4%) cumulatively belonged to the poorest, poorer, and middle wealth quintiles, and most women (66.2%) reported not working. More than two-thirds (69.2%) of the participants lived in rural areas. About half (50.2%) of the respondents were normal weight, while 31.7% and 14.0% were overweight and obese, respectively. The demographic characteristics of the participants are summarised in Table 1.

**Table 1:** Participants demographic characteristics

	Frequency	Percentage
*Education*		
No education	1.0	0.02
Primary	12	0.25
Secondary+	4780	99.7
*Wealth*		
Poorest	998	20.8
Poorer	991	20.7
Middle	1002	20.9
Richer	934	19.5
Richest	868	18.1
*Not working*		
Working	1617	33.8
Not working	3163	66.2
*BMI*		
Underweight	188	4.0
Normal weight	2383	50.2
Overweight	1,505	31.7
Obese	667	14.1
*Place of residence*		
Rural	3317	69.2
Urban	1476	30.8
*Age of households*		
15–24	850	17.8
25–34	1873	39.1
35–44	1436	30.0
45–54	634	13.2

As seen in Table 2, the percentage of individuals with hypertension was significantly higher among the 45–54 years age group (32%), and the prevalence of hypertension was significantly higher amongst all respondents with secondary and higher education (p < 0.0001). The hypertension prevalence was higher among the women from poor households (15.3% for poorest and 15% for poorer) than respondents from the middle wealth and rich households (11.5% for both middle and richer, 11.3% for the richest) (p < 0.0001). The prevalence of hypertension was higher among respondents who were not working (14.6%) than respondents in the working class (12%) (p < 0.0001). Additionally, the prevalence was higher among rural residents (13.6% vs. 11.4%) (p < 0.0001). Overweight (15.6) and obese (29.5%) participants had higher hypertension prevalence than underweight (3.7%) and normal weight (7.3%) participants.

**Table 2:** Participants demographic Characteristics association with hypertension

	Normotensive	Hypertensive	p-value*
	Percentage	Percentage	
*Education*			< 0.001
No education	100	0.0	
Primary	91.6	8.3	
Secondary+	87.0%	12.9	
*Wealth*			< 0.001
Poorest	84.7	15.3	
Poorer	85.0	15.0	
Middle	88.5	11.5	
Richer	88.5	11.5	
Richest	88.7	11.3	
*Not working*			< 0.001
Working	85.4	14.6	
Not working	88.0	12.0	
*Body mass index, kg/m*^*2*^			< 0.001
Underweight	96.3	3.7	
Normal weight	92.7	7.3	
Overweight	83.9	15.6	
Obese	70.4	29.5	
*Place of residence*			< 0.001
Rural	86.3	13.6	
Urban	88.6	11.4	
*Age of household members (years)*			< 0.001
15–24	96.2	3.8	
25–34	92.5	7.5	
35–44	83.0	17.1	
45–54	68.0	32.0	

*Statistically significant

Approximately (40.0%) of respondents reported to be hypertensive and were exposed to gender-based violence fell within the age group 35–44 years. In comparison, the prevalence was lower among 15–24 years (0.05%), followed by 25–34 (22.5%) years. Respondents with primary or no education were more likely to be exposed to GBV. Approximately all the respondents exposed to GBV and who were hypertensive had secondary or higher education (99.9% vs. 0.0%).

### Association between gender-based violence and hypertension

**Fig. 1:**
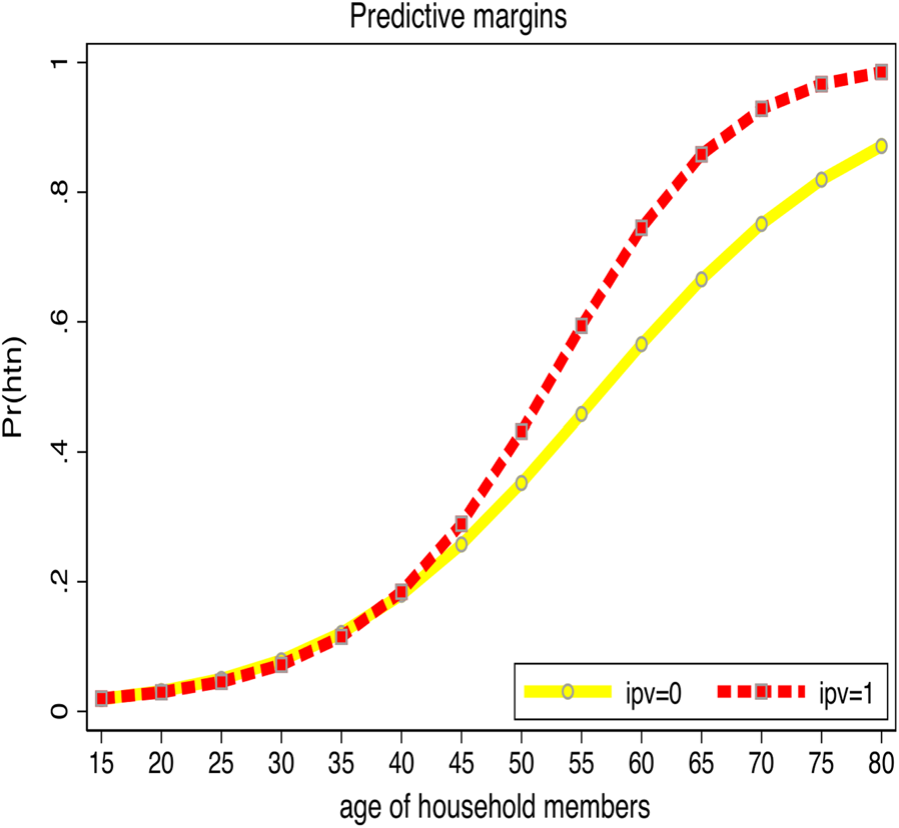
Age trajectories of hypertension (htn) among participants exposed to gender-based violence versus unexposed participants unexposed to gender-based violence. *IPV = 0 = unexposed to IPV, and IPV = 1 = exposed to IPV, *Pr = probability, *IPV = Intimate partner violence

Figure 1 describes the age trajectories of hypertension among participants exposed to GBV compared to participants not exposed to GBV. The odds of a higher prevalence of hypertension among those exposed to GBV increase with age. The difference was more pronounced after 45 to 80 years of age.

**Fig. 2:**
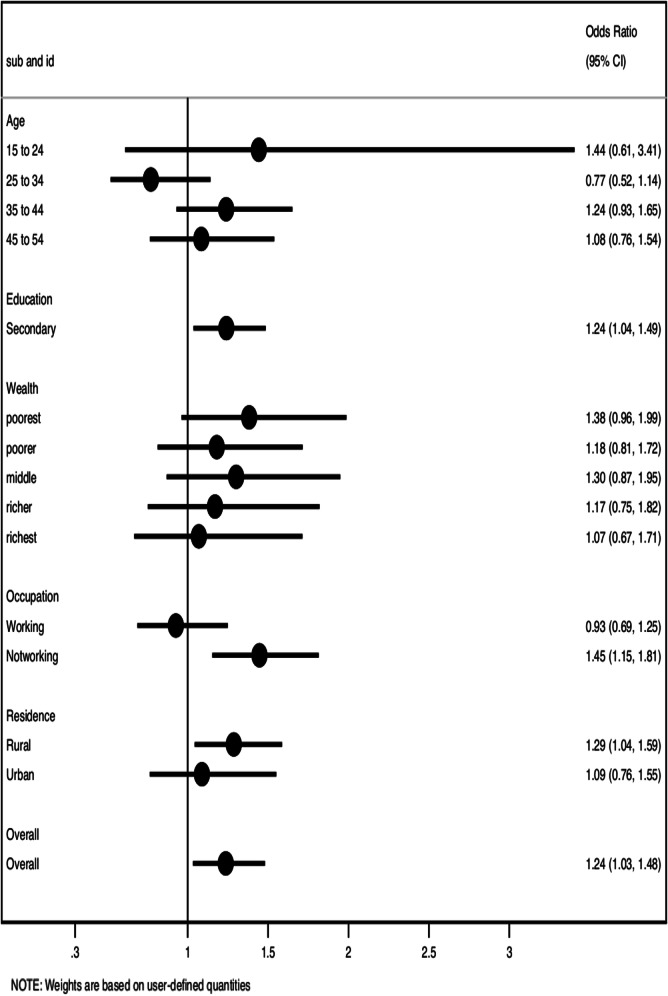
Association of gender-based violence with hypertension by subgroups

The measure of association between GBV and hypertension is summarised in Fig. 2. Overall, participants exposed to GBV were 24% more likely to have hypertension than those unexposed to GBV (OR = 1.24, 95% CI: 1.03–1.48), such that 621 (13%) of the total sample of 4793 had hypertension. Out of the 1401 exposed to GBV, 206 (14.7%) had hypertension, while 415 (12.2%) were not exposed to GBV but were hypertensive. Participants with secondary education or higher exposed to GBV were 24% more likely to have hypertension than those unexposed to GBV (OR = 1.24, 95% CI: 1.04–1.49). There was a significant association between hypertension and GBV among unemployed participants. Participants who were unemployed and exposed to GBV were 45% more likely to have hypertension than those not working and not exposed to GBV (OR = 1.45, 95% CI: 1.15–1.81). In addition, the association was only significant among rural residents. Rural residents exposed to GBV were 29% more likely to have hypertension than those unexposed to GBV (OR = 1.29, 95% CI: 1.04–1.59).

## Discussion

This study examined the association between GBV and hypertension among women in Kyrgyzstan. The findings showed that women exposed to GBV were more likely to have hypertension than GBV-unexposed women. The likelihood increased significantly among rural, highly educated, and unemployed women. These findings are consistent with previous studies. Clark et al. found that stress and poverty were significantly associated with GBV, and increased hypertension risk [19]. Another study on South African women revealed a positive association between intimate partner violence and hypertension [13]. Another study indicated that women’s exposure to GBV was associated with husbands’ age, education level, place of residence, low socioeconomic status and unemployment, which supports the findings of our study [20].

Jewkes. (2002) stated that poverty contributes more to IPV among people with lower socioeconomic status. Therefore, financial constraints make it difficult to access healthcare, which increases the risks of many diseases, including hypertension, for poor GBV victims. Moreover, financial stability protects against abuses at the individual level and is associated with lower acceptance of abuse and other forms of violence. Women’s equal or higher earnings and partner’s community support are controlling behaviours that decrease women’s vulnerability by 30–40%, underlining the importance of socioeconomic empowerment of women. On the other hand, it was found that employed women face a greater risk of abuse in communities with relatively higher acceptance of wife-beating [21], which may explain the higher risk of hypertension among highly educated women, probably from increased beating. Household wealth and women’s employment increase the likelihood of experiencing IPV among women in the Kyrgyz Republic. This is reinforced by Chernyak’s (2020) study, indicating that empowerment and experiential characteristics are risk factors for IPV. Therefore, there is an urgent need of empowering women and creating awareness among individuals with higher education, and high income and community education in the Kyrgyz Republic.

Our study also showed that the odds of having hypertension among women exposed to GBV increase with age, and the difference was more pronounced after 45–80 years of age. This aligns with Wet-Billings and Godongwana’s [13] findings that hypertension prevalence increased among older South African women. Age is a known risk factor for hypertension [22] and the more age advances, the more hypertension risk increases.

GBV remains one of the significant public health problems and has a severe impact on women’s health and wellbeing. Stress caused by violence perpetrated by a spouse or partner could be a confounding factor in developing hypertension in young women. Hypertension is costly as a chronic illness requiring medication and specialised dietary requirements. This study suggests that measures to eliminate abuse would also decrease hypertension incidences among young women in the Kyrgyzstan Republic, relieving both health care and financial burdens. Our study findings would help establish local programmes and policies to develop more holistic approaches to addressing young women’s care and health and social problems.

Future studies should investigate causality and see if GBV/IPV leads to hypertension later in life. In addition, qualitative research of GBV/IPV victims’ coping methods for managing hypertension would guide direct medical and social programmes to help these women.

### Study strengths and limitations

There are a few strengths of this study. The study focused on GBV and hypertension among women in the Kyrgyz Republic, using population-based data drawn from a large sample of the 2018 Kyrgyz Republic, which makes it more generalisable. This study was based on DHS reports whose data are widely perceived to be of high quality as they were based on a suitable sampling methodology with high response rates.

Despite the study’s strengths, some limitations should be considered when interpreting our findings. This study used secondary data, and therefore other confounding factors such as stress level could not be accounted for from the data. The time and period of abuse could not be determined, making it difficult to conclude that participants were hypertensive before the abuse. The study could not determine if the hypertension status of these young women was pregnancy-related, as is the case of pre or postpartum hypertension. The cross-sectional nature of the data limits its ability to draw causal inferences. The data employed an asset-based index as a proxy for household wealth status due to the unavailability of reliable income expenditure data.

## Conclusion

The study revealed that GBV is a significant problem in the Kyrgyzstan republic, contributing to increased hypertension risk among women. There is a need for more awareness of the impact and repercussions of GBV on the lives of individuals who experience any form of abuse, particularly among rural residents, those with secondary and higher education, and unemployed individuals. Therefore, we recommend a multi-sectorial approach to addressing interpersonal violence against women, girls, and children.

## Data Availability

The data supporting this article are available at: http://dhsprogram.com/data/available-datasets.cfm.

## References

[1] Zegenhagen S, Ranganathan M, Buller AM (2019). Household decision-making and its association with intimate partner violence: examining differences in men’s and women’s perceptions in Uganda. SSM-Popul Health.

[2] Cruz A, Klinger S: Gender-based violence in the world of work. 2016.

[3] Ahinkorah BO (2021). Polygyny and intimate partner violence in sub-Saharan Africa: evidence from 16 cross-sectional demographic and health surveys. SSM-Popul Health.

[4] UG Assembly (1948). Universal declaration of human rights. UN Gen Assem.

[5] General AU (2017). The United Nations universal declaration of human rights. Philos Now.

[6] Heise LL, Kotsadam A (2015). Cross-national and multilevel correlates of partner violence: an analysis of data from population-based surveys. Lancet Glob Health.

[7] World Health Organisation: Devastatingly pervasive: 1 in 3 women globally experience violence. 2021.

[8] Chowdhury R: Bride kidnapping: a deplorable custom repressing women in Kyrgyzstan. Available at SSRN 3786106. 2020.

[9] Cooper-Cunningham D: Domestic violence as everyday terrorism: bride kidnapping in Kyrgyzstan. Int Relat 2016.

[10] Sumner JA, Kubzansky LD, Roberts AL, Gilsanz P, Chen Q, Winning A (2016). Post-traumatic stress disorder symptoms and risk of hypertension over 22 years in a large cohort of younger and middle-aged women. Psychol Med.

[11] Gilbert L, Jiwatram-Negron T, Nikitin D, Rychkova O, McCrimmon T, Ermolaeva I (2017). Feasibility and preliminary effects of a screening, brief intervention and referral to treatment model to address gender-based violence among women who use drugs in Kyrgyzstan: project WINGS (Women Initiating New Goals of Safety). Drug Alcohol Rev.

[12] Akkina SK, Ricardo AC, Patel A, Das A, Bazzano LA, Brecklin C (2012). Illicit drug use, hypertension, and chronic kidney disease in the US adult population. Transl Res.

[13] Wet-Billings D, Godongwana M (2021). Exposure to intimate partner violence and hypertension outcomes among young women in South Africa. Int J Hypertens.

[14] Clark CJ, Alonso A, Everson-Rose SA, Spencer RA, Brady SS, Resnick MD (2016). Intimate partner violence in late adolescence and young adulthood and subsequent cardiovascular risk in adulthood. Prev Med.

[15] Chobanian AV (2006). Prehypertension revisited. Hypertension.

[16] Simpson L, Christensen A (2005). Spousal agreement regarding relationship aggression among treatment-seeking couples. Psychol Assess.

[17] Baba K, Takauma F, Tada K, Tanaka T, Sakanashi K, Kataoka Y (2017). Factor structure of the conflict tactics scale 1. Int J Commun Based Nursing Midwifery.

[18] Abba MS, Nduka CU, Anjorin S, Mohamed SF, Agogo E, Uthman OA (2021). Influence of contextual socioeconomic position on hypertension risk in low-and middle-income countries: disentangling context from composition. BMC Public Health.

[19] Clark MT, Wakesho KR, Bonsuk RS, Nabunya S, Kulaba BMK, Taylor J: Community-based safety partnerships to reduce gender-based violence in Uganda: the Anti-Domestic Violence and Abuse Center (ADOVIC) approach. In: The Routledge International Handbook of Domestic Violence and Abuse. Routledge, London; 2021. pp. 516-30.

[20] Sanjel S (2013). Gender-based violence: a crucial challenge for public health. Kathmandu Univ Med J.

[21] Cools S, Kotsadam A (2017). Resources and intimate partner violence in Sub-Saharan Africa. World Dev.

[22] Buford TW (2016). Hypertension and aging. Ageing Res Rev.

